# Publication Bias in Antipsychotic Trials: An Analysis of Efficacy Comparing the Published Literature to the US Food and Drug Administration Database

**DOI:** 10.1371/journal.pmed.1001189

**Published:** 2012-03-20

**Authors:** Erick H. Turner, Daniel Knoepflmacher, Lee Shapley

**Affiliations:** 1 Department of Psychiatry, Oregon Health & Science University, Portland, Oregon, United States of America; 2 Department of Pharmacology, Oregon Health & Science University, Portland, Oregon, United States of America; 3 Center for Ethics in Health Care, Oregon Health & Science University, Portland, Oregon, United States of America; 4 Behavioral Health and Neurosciences Division, Portland Veterans Affairs Medical Center, Portland, Oregon, United States of America; 5 School of Medicine, Oregon Health & Science University, Portland, Oregon, United States of America; Nordic Cochrane Centre, Rigshospitalet, Denmark

## Abstract

**Background:**

Publication bias compromises the validity of evidence-based medicine, yet a growing body of research shows that this problem is widespread. Efficacy data from drug regulatory agencies, e.g., the US Food and Drug Administration (FDA), can serve as a benchmark or control against which data in journal articles can be checked. Thus one may determine whether publication bias is present and quantify the extent to which it inflates apparent drug efficacy.

**Methods and Findings:**

FDA Drug Approval Packages for eight second-generation antipsychotics—aripiprazole, iloperidone, olanzapine, paliperidone, quetiapine, risperidone, risperidone long-acting injection (risperidone LAI), and ziprasidone—were used to identify a cohort of 24 FDA-registered premarketing trials. The results of these trials according to the FDA were compared with the results conveyed in corresponding journal articles. The relationship between study outcome and publication status was examined, and effect sizes derived from the two data sources were compared. Among the 24 FDA-registered trials, four (17%) were unpublished. Of these, three failed to show that the study drug had a statistical advantage over placebo, and one showed the study drug was statistically inferior to the active comparator. Among the 20 published trials, the five that were not positive, according to the FDA, showed some evidence of outcome reporting bias. However, the association between trial outcome and publication status did not reach statistical significance. Further, the apparent increase in the effect size point estimate due to publication bias was modest (8%) and not statistically significant. On the other hand, the effect size for unpublished trials (0.23, 95% confidence interval 0.07 to 0.39) was less than half that for the published trials (0.47, 95% confidence interval 0.40 to 0.54), a difference that was significant.

**Conclusions:**

The magnitude of publication bias found for antipsychotics was less than that found previously for antidepressants, possibly because antipsychotics demonstrate superiority to placebo more consistently. Without increased access to regulatory agency data, publication bias will continue to blur distinctions between effective and ineffective drugs.

*Please see later in the article for the Editors' Summary*

## Abstract 2

A comparison of data held by the U.S. Food and Drug Administration (FDA) against data from journal reports of clinical trials enables estimation of the extent of publication bias for antipsychotics.

## Editors' Summary

**Background:**

People assume that, when they are ill, health-care professionals will ensure that they get the best available treatment. But how do clinicians know which treatment is likely to be most effective? In the past, clinicians used their own experience to make such decisions. Nowadays, they rely on evidence-based medicine—the systematic review and appraisal of trials, studies that investigate the efficacy and safety of medical interventions in patients. Evidence-based medicine can guide clinicians, however, only if all the results from clinical trials are published in an unbiased manner. Unfortunately, “publication bias” is common. For example, the results of trials in which a new drug did not perform better than existing drugs or in which it had unwanted side effects often remain unpublished. Moreover, published trials can be subject to outcome reporting bias—the publication may only include those trial outcomes that support the use of the new treatment rather than presenting all the available data.

**Why Was This Study Done?:**

If only strongly positive results are published and negative results and side-effects remain unpublished, a drug will seem safer and more effective than it is in reality, which could affect clinical decision-making and patient outcomes. But how big a problem is publication bias? Here, researchers use US Food and Drug Administration (FDA) reviews as a benchmark to quantify the extent to which publication bias may be altering the apparent efficacy of second-generation antipsychotics (drugs used to treat schizophrenia and other mental illnesses that are characterized by a loss of contact with reality). In the US, all new drugs have to be approved by the FDA before they can be marketed. During this approval process, the FDA collects and keeps complete information about premarketing trials, including descriptions of their design and prespecified outcome measures and all the data collected during the trials. Thus, a comparison of the results included in the FDA reviews for a group of trials and the results that appear in the literature for the same trials can provide direct evidence about publication bias.

**What Did the Researchers Do and Find?:**

The researchers identified 24 FDA-registered premarketing trials that investigated the use of eight second-generation antipsychotics for the treatment of schizophrenia or schizoaffective disorder. They searched the published literature for reports of these trials, and, by comparing the results of these trials according to the FDA with the results in the published articles, they examined the relationship between the study outcome (did the FDA consider it positive or negative?) and publication and looked for outcome reporting bias. Four of the 24 FDA-registered trials were unpublished. Three of these unpublished trials failed to show that the study drug was more effective than a placebo (a “dummy” pill); the fourth showed that the study drug was inferior to another drug already in use in the US. Among the 20 published trials, the five that the FDA judged not positive showed some evidence of publication bias. However, the association between trial outcome and publication status did not reach statistical significance (it might have happened by chance), and the mean effect size (a measure of drug effectiveness) derived from the published literature was only slightly higher than that derived from the FDA records. By contrast, within the FDA dataset, the mean effect size of the published trials was approximately double that of the unpublished trials.

**What Do These Findings Mean?:**

The accuracy of these findings is limited by the small number of trials analyzed. Moreover, this study considers only the efficacy and not the safety of these drugs, it assumes that the FDA database is complete and unbiased, and its findings are not generalizable to other conditions that antipsychotics are used to treat. Nevertheless, these findings show that publication bias in the reporting of trials of second-generation antipsychotic drugs enhances the apparent efficacy of these drugs. Although the magnitude of the publication bias seen here is less than that seen in a similar study of antidepressant drugs, these findings show how selective reporting of clinical trial data undermines the integrity of the evidence base and can deprive clinicians of accurate data on which to base their prescribing decisions. Increased access to FDA reviews, suggest the researchers, is therefore essential to prevent publication bias continuing to blur distinctions between effective and ineffective drugs.

**Additional Information:**

Please access these web sites via the online version of this summary at http://dx.doi.org/10.1371/journal.pmed.1001189.

- The US Food and Drug Administration provides information about drug approval in the US for consumers and health-care professionals
- Detailed information about the process by which drugs are approved is on the web site of the FDA Center for Drug Evaluation and Research; also, FDA Drug Approval Packages are available for many drugs; the FDA Transparency Initiative, which was launched in June 2009, is an agency-wide effort to improve the transparency of the FDA
- FDA-approved product labeling on drugs marketed in the US can be found at the US National Library of Medicine's DailyMed web page
- Wikipedia has a page on publication bias (note: Wikipedia is a free online encyclopedia that anyone can edit; available in several languages)
- MedlinePlus provides links to sources of information on schizophrenia and on psychotic disorders (in English and Spanish)
- Patient experiences of psychosis, including the effects of medication, are provided by the charity HealthtalkOnline

## Introduction

Evidence-based medicine is valuable only to the extent that the available evidence is complete and unbiased. Unfortunately, whether research results are published, and how they are published, often depends on their statistical significance [1],[2], which alters the apparent risk–benefit ratio of drugs. Within medicine, a recent review found evidence for various forms of publication bias within 40 different indications [3].

Despite ample evidence for the existence of publication bias, there is little evidence of its quantitative impact on the apparent efficacy of most drugs. Most methods for studying publication bias provide indirect evidence for nonpublication or outcome reporting bias. A frequently used method is to examine a cohort of published studies for “small study effects” (smaller studies showing larger treatment effects) in the form of funnel plot asymmetry [4]. Despite its wide use, this approach has limitations. First, although a funnel plot may suggest that studies with smaller effect sizes have not been published, it cannot prove that such studies in fact ever existed. Second, the effect sizes plotted are based on results that are published, and one cannot be sure whether and to what extent such results have been affected by outcome reporting bias. The true underlying results—the results according to the prespecified outcomes—usually remain unknown.

By contrast, such data are often available from drug regulators, such as the US Food and Drug Administration (FDA). Because the FDA gathers data from premarketing trials both before inception and after completion, it functions as both a registry and a results database [5]. For any given cohort of trials, results according to FDA reviews can be compared to corresponding results according to the published literature. Any discrepancies between the two sources provide direct evidence of publication bias.

FDA reviews have been used to document publication bias across various medical indications in at least two studies [6],[7]. An advantage of looking broadly across medical indications is that it documents the wide scope of publication bias, while focusing on a single indication may lead some readers to assume that the phenomenon is specific to that indication. On the other hand, FDA data from a single indication may be more useful to meta-analysts, since data measuring the same construct allow for the calculation of an overall effect size. Such data should also be useful to clinicians, who are interested in the true efficacy of a specific drug class that they prescribe.

In a previous study of antidepressants, our group found that publication bias nearly doubled the apparent proportion of positive trials and increased the apparent effect size by one-third [8]. This raises the question as to whether publication bias similarly affects the apparent efficacy of other drug classes.

Schizophrenia has a lifetime prevalence of 0.55% [9], and its core symptom, psychosis, is the third most disabling condition worldwide [10]. The objective of this study is to use FDA data as an independent benchmark or control to determine whether, and to what extent, the apparent efficacy of second-generation antipsychotics has been influenced by publication bias.

## Methods

### Ethics Statement

This study was approved by the Research and Development Committee of the Portland Veterans Affairs Medical Center. It was reported according to the guidelines of the Preferred Reporting Items for Systematic Reviews and Meta-Analyses (PRISMA) group (Text S1).

### Data Procurement

#### Procurement of data from FDA reviews

We identified the phase 2/3 clinical trial programs leading to the FDA's marketing approval of eight second-generation antipsychotic drugs for the treatment of schizophrenia. This retrospective cohort consisted of 24 FDA-registered short-term double-blind placebo-controlled trials conducted between December 1993 and May 2005. The FDA Drug Approval Packages were publicly available at, and downloaded from, the FDA's web site [5],[11] for all drugs except risperidone (Risperdal), for which we obtained the Drug Approval Package via request to the FDA's Freedom of Information Office. (Because risperidone was approved in 1993, it was not subject to the Electronic Freedom of Information Act of 1996 [12].) Within the Drug Approval Packages, we examined data relevant to the agency's determination of drug efficacy in medical reviews, statistical reviews, and administrative correspondence.

In order to make these documents more accessible to readers and researchers, the FDA Drug Approval Package documents were processed using Adobe Acrobat as follows: (1) reviews of the same type but presented as multiple PDF files (e.g., Medical Review Parts 1, 2, 3, and 4) were combined into single PDFs; (2) page numbers were added as footers; (3) because the FDA had presented the documents in an unsearchable format [5], they were rendered searchable using optical character recognition; and (4) text directly quoted in the present article was highlighted. These documents have been placed in a digital repository of Oregon Health & Science University. The reader may find them by navigating to (1) http://www.ohsu.edu/library/, (2) “Digital Resources Library,” then (3) “FDA Drug Approval Documents.”

#### Procurement of data from journal articles (literature search)

The published literature was searched for journal articles matching each FDA-reviewed trial, with a cutoff date of May 5, 2010. The best match for each trial was identified using the following parameters: drug name, name of active comparator (when used), dosage groups, their sample sizes, trial duration, and name of principal investigator.

The initial searches employed two databases, PubMed and Cochrane Central Register of Controlled Trials. The search strategy was for the title field to include the name of the drug and either “schizophrenia” or “schizoaffective,” and for the word “placebo” to appear in any field (e.g., title, keywords, abstract). As an example, when searching Cochrane Central Register of Controlled Trials for relevant aripiprazole trials, the search syntax was “(aripiprazole and schizo$).ti. and placebo.af.”

From the search output, titles and abstracts were screened so as to exclude journal articles focused on topics other than the overall efficacy of the drug for schizophrenia or schizoaffective disorder. Thus, articles focused on the following topics were excluded: other indications (e.g., bipolar disorder, treatment-resistant schizophrenia), subsets with specific comorbid conditions, particular symptom clusters (e.g., agitation, weight change), safety (as opposed to efficacy), specific demographic samples, trials lacking a parallel design (add-on, open-label, crossover), trials that were not placebo-controlled, trials not involving acute treatment (long-term trials, including maintenance trials), and trials involving other routes of administration.

Trials were counted as published according to the method of our earlier study [8]. The intent was to include journal articles that provided data sufficient for meta-analysis yet were reasonably discoverable by, and accessible to, the average clinician. Articles in languages other than English were excluded. Meeting abstracts were excluded—trials were required to be fully published [13]. An FDA-registered trial was considered published if it could be matched with a primary publication [14]. The preferred type of primary publication was a stand-alone publication (i.e., an article devoted to reporting the results of a single trial). If no stand-alone publication could be found, aggregate publications, in which multiple trials were covered in a single article, were sought. However, aggregate publications were accepted only if all trials included in the article presented primary data; aggregate publications that were heterogeneous, i.e., those reporting a mix of primary and secondary trial data, were excluded.

If the original searches using PubMed and the Cochrane Central Register of Controlled Trials suggested that an FDA-registered trial was unpublished, Ovid Medline was searched for the three most recent review articles focused on the efficacy of the drug in question. These review articles were then examined for trial bibliographic information. The Ovid Medline search strategy was similar to that given above for the Cochrane Central Register of Controlled Trials, except that the search was restricted to review articles in English and that “placebo” was omitted as a search term. For example, when the original search yielded no journal articles matching two FDA-registered trials of aripiprazole, the following search strategy was used: (1) “(aripiprazole and schizo$).ti. and review.pt.”; (2) limit (1) to English language.

Finally, because pharmaceutical companies pledged in 2004 to increase transparency by posting trial results publicly, the PhRMA Clinical Study Results Database (decommissioned December 2011) and the drug sponsors' own web sites were searched for information as to whether these trials were published.

If all the above steps yielded no evidence of publication, it was concluded that that the trial in question was not reasonably accessible to the average clinician, and it was considered unpublished.

### Data Extraction and Entry

Double data extraction and entry was employed in this study. The primary outcome was identified for each trial by two authors (D. K. and L. S.) working independently, with the stipulation that it be either the Brief Psychiatric Rating Scale (BPRS) [15] or the Positive and Negative Syndrome Scale (PANSS) [16]. The two raters' sets of entries were compared, and any discrepancies were resolved through consensus. The primary rating scale according to the FDA matched that in the journal article for all trials but one.

Throughout the FDA reviews and journal articles, the primary analyses involved modified intention-to-treat methods [17] for handling dropouts: mixed-effects model repeated measures [18] was used for some of the iloperidone trials (in both FDA reviews and journal articles); last observation carried forward (LOCF) [19] was used for all other trials. The numerical results according to the primary outcome (*p*-values, means, standard deviations, standard errors, and/or confidence intervals) were extracted and entered independently by D. K. and L. S., for the FDA data followed by the journal article data. Boolean formulas in Excel were used to compare and flag any mismatches between the two sets of entries. For each mismatch, the original data source (FDA review or journal article) was re-examined by E. H. T. to determine which of the two entries was correct.

In addition to the numerical or continuous data mentioned above, categorical data were extracted. The FDA's regulatory decision on each trial was rated independently by D. K. and L. S. at one of three levels: positive (supportive of efficacy), questionable (neither clearly positive nor clearly negative), or negative (not supportive of efficacy). Any discrepancies between the two sets of ratings were discussed among the three authors while consulting the FDA review materials, and consensus was reached. When the judgment in the FDA's review was unclear, the clinical trials section of the original product labeling was downloaded from the FDA web site and referred to for clarification. For each journal publication, the presentation of the result on the primary outcome was rated, again independently by D. K. and L. S., as positive, negative, or questionable. Again, any differences between the two sets of ratings were resolved by consensus.

For trials where the FDA ratings and journal ratings disagreed, the authors shared and discussed their observations as to how they disagreed. In contrast to our previously published study [8], the differences found were, we felt, too varied and nuanced to be meaningfully and reliably captured using a categorical rating system and then subjected to statistical analysis. Instead, the differences between the FDA and journal presentations of the trial results are described in narrative format. This text was drafted by E. H. T., critically revised by D. K. and L. S., and includes several direct quotes from the data sources.

### Data Analysis

#### Trial outcome versus publication status

As noted above, the FDA's regulatory decisions regarding the trials were classified in this study as (1) positive (clearly supportive of efficacy), (2) questionable (marginal or borderline support for efficacy), or (3) negative (clearly not supportive of efficacy). Categories 2 and 3 were combined into a not-positive grouping. The strength of the association between the FDA regulatory decision and publication status (published versus unpublished) was calculated as Fisher's exact *p* (two-tailed) using the csi command with the exact option in Stata 11 [20].

#### Meta-analysis

As described previously [8], two meta-analyses were conducted: a conventional meta-analysis using published data, and a control meta-analysis using FDA data. Data from active comparators were excluded, so that each drug's effect size was derived solely from data collected by that drug's sponsor. The measure of effect size used was Hedges's *g* [21], calculated using the following equation [22]:
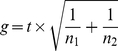
(1)The values for *g* were adjusted using Hedges's correction for small sample size [23]. To calculate *t*, as previously described [8], precise *p*-values were used together with the degrees of freedom as arguments in Excel's TINV function. If the *p*-value was instead reported as a range (e.g., “*p*<0.05”), collateral data to calculate effect size were used according to the following hierarchy: standard deviations, standard errors, and 95% confidence interval around the mean difference. In the few cases where none of these data were available in the journal article, data were imputed from the FDA database. Conversely, when the FDA database lacked the necessary data, data were imputed from the corresponding journal article. The purpose of this imputation was to err in the direction of the null hypothesis of no difference in effect size between the FDA and the published literature. By convention, positive and negative effect size values were used to signify superiority and inferiority to placebo, respectively.

The typical trial compared multiple doses of study drug to placebo (fixed-dose design). In such cases, a single trial-level effect size and standard error was calculated using a fixed effects model [24] to pool the values from that trial's multiple treatment arms. To avoid a spuriously low standard error, each trial's shared placebo *n* was counted once rather than redundantly for each dose group. A limitation of this method is that it only partially addresses error due to correlation between the comparisons [25].

Using the trial-level effect size values, the random effects pooling method [26] in Stata [20],[27] was used to calculate mean weighted values for each drug and for the entire drug class. Heterogeneity was assessed using the *I* ^2^ statistic [28]. As has been recommended [29], 95% confidence intervals around *I* ^2^ were calculated using the non-central chi-squared-based approach within the heterogi module [30] for Stata.

As an error check, author E. H. T. returned to the dataset from the original double data extraction and entry, repeated the calculations, and electronically compared the resulting values with those calculated earlier.

Meta-regression was conducted using the Metareg module for Stata 11 [27] to contrast the FDA-based effect sizes of the published trials with those of the unpublished trials. The method used for estimating the between-study variance was residual maximum likelihood, the default method in Stata 11. A similar analysis was used to contrast the effect sizes derived from the FDA data, from both published and unpublished trials, with the effect sizes derived from the published literature.

## Results

### Number of Trials

According to the FDA reviews, the eight drugs examined were approved based on the efficacy results from 24 short-term double-blind placebo-controlled trials. Thus, three trials were required, on average, to approve one second-generation antipsychotic. Table 1 shows the characteristics of these trials according to the FDA. Table 2 shows bibliographic information on these trials. As seen, 20 of the trials (83%) were published and four (17%) were unpublished. The 20 published trials were published in 19 journal articles. The lack of one-to-one correspondence occurred because (1) one article [31] reported the results of three iloperidone trials (included because their results were not reported elsewhere in stand-alone form—see Methods), and (2) one FDA-registered trial of risperidone was published in two separate articles, one for the Canadian sites and another for the US sites, as if they were separate trials (details below).

**Table 1:** Characteristics of premarketing trials of antipsychotics according to FDA.

Drug Being Studied	Trial Number or Identifier	Number of Sites (Location)	Diagnoses Included		Treatment Arm			Duration (wks)	Primary Rating Scale	Baseline Score
			Scz	Scz-Aff	Drug Group	Dose (mg)	*n* Analyzed			
**Aripiprazole (Abilify)**	93202	10 (US)	+	−	Aripiprazole	30	33	4	BPRSd	53
					Haloperidol	20	33			50.3
					Pbo	0	35			50
	94202	22 (US)	+	−	Aripiprazole	10	51	4	BPRS core	17
						30	54			16.2
					Haloperidol	10	54			16.6
					Pbo	0	57			16.1
	97201	36 (US)	+	+	Aripiprazole	15	99	4	PANSS	98.8
						30	100			99.6
					Haloperidol	10	99			99.9
					Pbo	0	102			100.9
	97202	40 (US)	+	+	Aripiprazole	20	98	4	PANSS	94
						30	96			92.3
					Risperidone	6	95			93.6
					Pbo	0	103			95
	138001	57 (US, Canada)	+	−	Aripiprazole	10	103	6	PANSS	92.76
						15	103			93.27
						20	97			92.29
					Pbo	0	107			92.4
**Iloperidone (Fanapt)**	3000	45 (US)	+	+	Iloperidone	12	115 (82)	6	PANSS	94.6
					Haloperidol	15	115 (70)			96.1
					Pbo	0	117 (78)			95
	3004	65 (No Am, So Afr, Eur, Australia)	+	+	Iloperidone	10–16	149 (121)	6	BPRSd	54.1
					Risperidone	4–8	146 (110)			54.7
					Pbo	0	152 (116)			54.2
	3005	67 (No Am, So Afr, Isr, Eur)	+	+	Iloperidone	12–16	230 (178)	6	BPRSd	54.4
						20–24	141 (111)			54.9
					Risperidone	6–8	148 (119)			55
					Pbo	0	152 (113)			55.4
	3101	44 (US, India)	+	−	Iloperidone	24	283	4	PANSS	92.88
					Ziprasidone	160	144			90.95
					Pbo	0	140			90.48
**Olanzapine (Zyprexa)**	HGAD	23 (US, Canada)	+	−	Olanzapine	10	62	6	BPRS	42.84
						15	65			42.62
					Haloperidol	15	68			41.79
					Pbo	0	62			39.69
	HGAP	12 (US)	+	−	Olanzapine	10	49	6	BPRS	37.43
					Pbo	0	49			36.78
**Paliperidone (Invega)**	303	53 (Eastern and Western Eur)	+	−	Paliperidone	6	123	6	PANSS	94.3
						9	122			93.2
						12	129			94.6
					Pbo	0	126			94.1
	304	45 (US)	+	−	Paliperidone	6	110	6	PANSS	92.3
						12	111			94.1
					Olanzapine	10	105			—
					Pbo	0	105			93.6
	305	74 (No Am, Eastern Eur, Asia, Isr, Mex, So Afr)	+	−	Paliperidone	3	123	6	PANSS	91.6
						9	123			93.9
						15	113			92.4
					Olanzapine	10	—			—
					Pbo	0	120			93.9
**Quetiapine (Seroquel)**	0001/0008	37 (US, Eur)	+	−	Quetiapine	≤250	92	6	BPRS	38.89
						≤750	94			41.04
					Pbo	0	94			38.35
	0006	11 (US)	+	−	Quetiapine	75–750	53	6	BPRS	55.79
					Pbo	0	53			54.09
	0013	26 (US, Canada)	+	−	Quetiapine	150	48	6	BPRS	47.15
						300	51			45.29
						600	51			43.45
						750	53			45.72
					Haloperidol	12	50			44
					Pbo	0	51			45.31
**Risperidone (Risperdal)**	201	9 (US)	+	−	Risperidone	1–10	51	6	BPRS	56.2
					Haloperidol	20	52			53.1
					Pbo	0	53			52.8
	204	28 (US, Canada)	+	−	Risperidone	6	85	8	PANSS	94.9
						10	85			91.8
						16	85			93.9
					Haloperidol	20	85			93.6
					Pbo	0	86			92.6
**Risperidone LAI (Consta)**	RIS-USA-121	47 (US)	+	−	Risperidone LAI	25	93	12	PANSS	81.7
						50	98			82.3
					Pbo	0	92			82
**Ziprasidone (Geodon)**	104	17 (US)	+	+	Ziprasidone	40	55	4	BPRS	34.5
						80	47			36.2
					Pbo	0	47			33.4
	106	12 (US)	+	+	Ziprasidone	40	43	4	BPRS	36.5
						120	41			36.6
					Pbo	0	47			37
	114	34 (US, Canada)	+	+	Ziprasidone	80	104	6	BPRSd	56.5
						160	103			55
					Pbo	0	91			55.1
	115	54 (US)	+	+	Ziprasidone	40	86	6	BPRSd	53.8
						120	76			51.8
						200	82			51.8
					Haloperidol	15	82			53.9
					Pbo	0	80			54.3

Drug brand name shown in parentheses. A dash indicates that the baseline score is not available in the FDA review. For aripiprazole Trial 94202, the number of sites is based on FDA analysis, which excluded site #003 (see text). For the first three iloperidone trials, parentheses enclose the sample size of the subset of patients with schizophrenia analyzed by the FDA. Dose groups shown only if within FDA-approved dose range. For paliperidone Trials 304 and 305, the FDA review did not list results for the olanzapine group: the corresponding *n* was taken from the corresponding journal article.

BPRSd, BPRS score derived from PANSS score; Eur, Europe; Isr, Israel; Mex, Mexico; No Am, North America; Pbo, placebo; So Afr, South Africa; Scz, schizophrenia; Scz-Aff, schizoaffective disorder.

**Table 2:** Bibliographic information on FDA-registered antipsychotic trials.

Sponsor	Drug Name	Trial Number or Identifier	First Author	Year	Journal Name	PubMed ID	Reference
**AstraZeneca**	Quetiapine	0006	Borison	1996	J Clin Psychopharmacol	8690831	[35]
		0001/0008	Small	1997	Arch Gen Psychiatry	9193196	[57]
		0013	Arvanitis	1997	Biol Psychiatry	9270900	[58]
**BMS**	Aripiprazole	93202	**Unpublished**				
		94202	**Unpublished**				
		97201	Kane	2002	J Clin Psychiatry	12363115	[59]
		97202	Potkin	2003	Arch Gen Psychiatry	12860772	[60]
		138001	McEvoy	2007	J Psychiatr Res	17631314	[61]
**Janssen**	Paliperidone	303	Kane	2007	Schizophr Res	17092691	[62]
		304	Marder	2007	Biol Psychiatry	17601495	[63]
		305	Davidson	2007	Schizophr Res	17466492	[64]
	Risperidone	201	Borison	1992	Psychopharmacol Bull	13811002	[37]
		204 (US sites)	Marder	1994	Am J Psychiatry	7514366	[41]
		204 (Canadian sites)	Chouinard	1993	J Clin Psychopharmacol	7683702	[40]
	Risperidone LAI	RIS-USA-121	Kane	2003	Am J Psychiatry	12777271	[65]
**Lilly**	Olanzapine	HGAD	Beasley	1996	Neuropsychopharmacology	8822534	[66]
		HGAP	Beasley	1996	Psychopharmacology (Berl)	8935812	[67]
**Novartis/Vanda**	Iloperidone	3000	Potkin	2008	J Clin Psychopharmacol	18334911	[31]
		3004					
		3005					
		3101	Cutler	2008	J Clin Psychopharmacol	18334909	[34]
**Pfizer**	Ziprasidone	104	**Unpublished**				
		106	Keck	1998	Psychopharmacology (Berl)	9860108	[36]
		114	Daniel	1999	Neuropsychopharmacology	10192829	[68]
		115	**Unpublished**				

### Trial Outcome versus Publication Status

**Figure 1:**
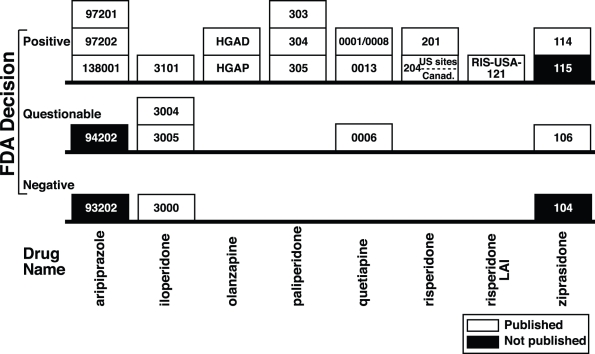
Trial outcome according to the FDA versus publication status of 24 premarketing trials of eight second-generation antipsychotics. Published trials are shown as white boxes, unpublished trials are shown as black boxes, and the trial numbers are shown within the boxes. Unpublished trials were, with one exception, not positive (either negative or questionable). Please see the text for narrative descriptions of several of these trials. Please note that the number of white boxes does not equal the number of journal articles (see text and Table 2): iloperidone trials 3000, 3004, and 3005 were published in a single journal article, and risperidone trial 204 was published as two separate journal articles, as though the US and Canadian sites constituted two distinct positive trials.

While most of the published trials (15/20 = 75%) were FDA-positive, most of the unpublished trials (3/4 = 75%) were FDA-negative or -questionable. The relationship between trial outcome and publication status, shown in Figure 1, did not reach statistical significance (Fisher's exact test *p* = 0.09). Handling the FDA-questionable category in alternate ways yielded similar *p-*values (see Table S1). In a post hoc analysis conducted after reclassifying unpublished ziprasidone Trial 115 from positive to questionable (in part because of its statistical inferiority to the active comparator haloperidol—see narrative trial details below), this relationship did reach statistical significance (*p* = 0.012). When this trial was excluded from the analysis, it remained significant (*p* = 0.03).

Overall, according to the FDA reviews, two-thirds of the trials (16/24 = 67%, binomial 95% confidence interval [CI_95%_] 45% to 84%) were positive, with the remaining one-third either questionable or negative. By contrast, according to the journal articles, there were 21 trials, all of them positive (CI_95%_ 84% to 100%).

### Meta-Analysis

**Figure 2:**
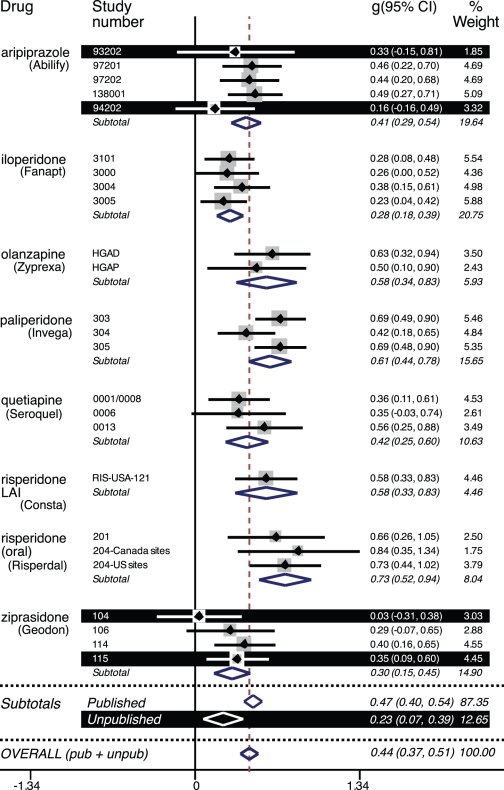
Forest plot of effect size (Hedges's *g*) values for FDA-registered premarketing trials of second-generation antipsychotics. The black horizontal bars highlight the four unpublished trials, three of which demonstrated no statistically significant advantage for the drug over placebo. The fourth unpublished trial showed that the drug was superior to placebo but statistically inferior to the active comparator haloperidol. Below the first dotted horizontal line, subtotal effect size and confidence interval values are shown for the subset of published trials and for the subset of unpublished trials. The difference in effect size between the published and unpublished trials was statistically significant (see text). Below the second dotted horizontal line, the FDA-based effect size is shown for all trials combined (0.44). The effect sizes shown for iloperidone trials 3000, 3004, and 3005 are based on the full sample of patients recruited, i.e., those with either schizophrenia or schizoaffective disorder, as opposed to the subset of patients with schizophrenia only (see text for details).

The dose groups reported on in the journal articles matched those in the FDA reviews. The statistical output from the two meta-analyses, i.e., that of the FDA and that of the journal data, is reproduced in Table S2 and Table S3, respectively. Figure 2 is a forest plot of the effect size and confidence intervals based on the data from the FDA reviews. For the published trials, the effect size was 0.47 (CI_95%_ 0.40 to 0.54; *I* ^2^ 38%, CI_95%_ 0% to 62%). For the unpublished trials, the effect size was less than half that, 0.23 (CI_95%_ 0.07 to 0.39; *I* ^2^ 0%, CI_95%_ 0% to 68%). By meta-regression, the difference between the effect sizes for the published versus unpublished trials was statistically significant (β = −0.25, CI_95%_−0.47 to −0.03, *t* = −2.36, *p* = 0.027). Because the unpublished trials were confined to two of the eight drugs, this latter analysis was repeated while adding drug as an explanatory variable. Here the effect of drug was not statistically significant (β = 0.02, CI_95%_ −0.01 to 0.05, *t* = 1.35, *p* = 0.19), and the difference between the published and unpublished trials remained significant (β = −0.28, CI_95%_ −0.49 to −0.06, *t* = −2.62, *p* = 0.016).

Within the published trials, there was essentially no difference between the effect sizes derived from the FDA reviews (0.47 from above) and those derived from the journal articles (0.48, CI_95%_ 0.40 to 0.56; *I* ^2^ 46%, CI_95%_ 0% to 66%). By meta-regression, this difference was not significant (β = 0.002, CI_95%_ −0.11 to 0.11, *t* = 0.03, *p* = 0.98).

**Figure 3:**
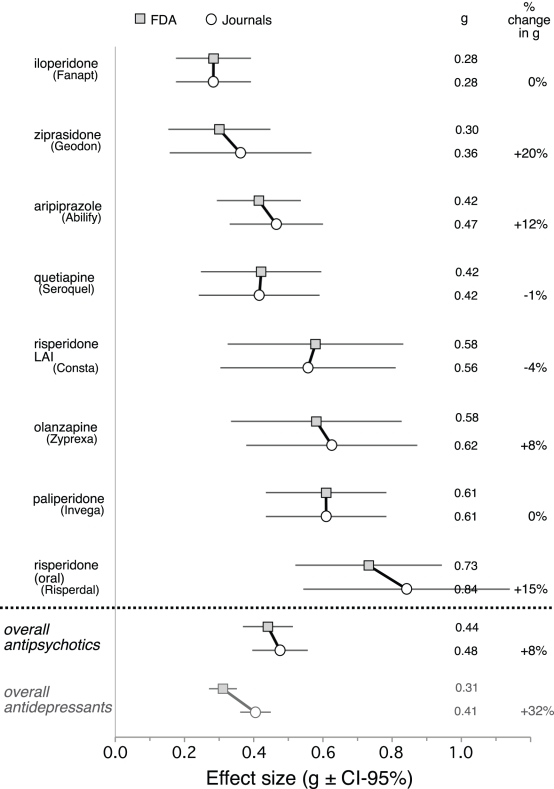
Forest plot of mean weighted effect size (Hedges's *g*) values by drug and data source. The figure is sorted according to the effect size point estimates derived from the FDA data; these are shown with gray-filled squares. The point estimates derived from the corresponding journal articles are shown with open circles. Horizontal lines indicate 95% confidence intervals around the effect size point estimates. The right-hand column shows the percent differences between the FDA-based and the journal-based effect size point estimates. (These percent differences are based on estimates accurate to three decimal places, while the estimates shown are rounded to two decimal places.) The differences ranged from −4% to +20%, depending on the drug. For the entire drug class (labeled “overall antipsychotics”), publication bias increased apparent effect size by 8%. To provide context, previously published data on antidepressants [8] are shown at the bottom in gray. Note that the 8% increase with antipsychotics is much less than the 32% increase with antidepressants [8]. Also, the journal data show only a 16% advantage in effect size for antipsychotics over antidepressants (from 0.41 to 0.48), while FDA data reveal a much larger advantage (42%, from 0.31 to 0.44).

The FDA data from the published and unpublished trials were combined into overall FDA-based effect sizes for each of eight second-generation antipsychotics. These effect sizes, along with their confidence intervals, are shown alongside their corresponding journal-based effect sizes in Figure 3. For individual drugs, the difference in effect size ranged from a 4% decrease (risperidone LAI) to a 20% increase (ziprasidone). With all drugs combined, the overall FDA-based effect size was 0.44 (CI_95%_ 0.37 to 0.51; *I* ^2^ 43%, CI_95%_ 0% to 63%). Compared to this, the overall journal-based effect size (0.48 from above) represented a slight (8%) increase in effect size due to publication bias, which was nonsignificant by meta-regression (β = 0.03, CI_95%_ −0.08 to 0.14, *t* = 0.62, *p* = 0.54). For each of the four effect sizes reported above, the lower confidence limit for *I* ^2^ was zero, and the upper confidence limit ranged from 62% to 68%, between proposed landmarks for moderate and high levels of heterogeneity [32].

### Unpublished Trials (*n* = 4 Trials)

There were four trials for which we were unable to find any evidence of publication. The literature search efforts are detailed for each trial in Text S2. Following are details regarding the conduct and results of these trials.

#### Unpublished aripiprazole trials (*n* = 2)

Aripiprazole Trial 94202 was one of two unpublished aripiprazole trials (Table 2). As shown in Table 1, it involved over 200 patient-participants at 22 sites in the US. According to the FDA medical officer review (pages 43 and 168 of 238), the data from one of the sites (site 003) were excluded because its investigator (Richard Borison, M.D., Ph.D.) was “disqualified due to allegations of research misconduct and conviction on criminal charges.” Neither the 10-mg dose nor the 30-mg dose separated from placebo (*p* = 0.89 and *p* = 0.12, respectively). The active comparator haloperidol separated from placebo on one of the primary rating scales (BPRS core, *p* = 0.0495) but not the other (Clinical Global Impression Scale–Improvement, *p* = 0.08). Consistent with the former, the statistical reviewer deemed the trial negative; consistent with the latter, the medical reviewer deemed the trial “failed.” For our purposes, we adopted the more conservative judgment of the study as failed, and thus classified it as questionable (Figure 1).

Aripiprazole Trial 93202 involved over 100 patients at ten US sites (Table 1). Like aripiprazole Trial 94202, aripiprazole did not separate statistically from placebo (*p* = 0.173), but unlike Trial 94202, the active comparator haloperidol clearly separated from placebo (*p* = 0.010). Thus, the FDA deemed Trial 93202 a negative, rather than a failed, trial (Figure 1). This trial was not published.

#### Unpublished ziprasidone trials (*n* = 2)

Ziprasidone Trial 115 was one of two unpublished ziprasidone trials (see Text S2 for results of literature search). As seen in Table 1, ziprasidone Trial 115 involved 53 US sites and over 400 patients and five treatment arms: three doses of ziprasidone (20 mg bid, 60 mg bid, and 100 mg bid), placebo, and haloperidol 10 mg as an active comparator. All active treatment arms demonstrated statistical superiority to the placebo arm, and there was evidence of a positive dose–response relationship. Because the FDA judges efficacy based on whether the study drug demonstrates superiority to placebo, the trial was ultimately considered positive.

Earlier, however, efficacy results from this trial, together with a safety concern, proved to be a temporary obstacle to marketing approval. When ziprasidone was first being reviewed, the FDA was concerned about the drug's tendency to prolong the QT interval and how that might affect the overall risk–benefit ratio and, consequently, its approvability. The cardiology consultant is quoted within the medical review (page 2 of 223):

Dr. Ganley recommended that, “unless efficacy data suggests superior benefit over currently available drugs, ziprasidone should be considered for second line therapy with adequate warnings of risk associated with drugs that prolong the QT interval.”

Ziprasidone Trial 115 was the only premarketing trial in which ziprasidone was compared to a marketed antipsychotic as well as to placebo. In this trial, ziprasidone failed to show the above-mentioned “superior benefit over currently available drugs.” In fact, it showed the opposite, that ziprasidone was statistically inferior to the active comparator haloperidol. According to the statistical review (page 7 of 61):

The haloperidol (active control) seemed to have a larger decrease in changes from baseline for most of the primary efficacy endpoints.

This statement was followed in the statistical review by Table 1R, which documented the statistical superiority of haloperidol over ziprasidone in this trial. In the column comparing the combination of all three ziprasidone dose groups to haloperidol, the *p-*values achieved on the scales BPRS total, BPRS core, Clinical Global Impression Scale–Severity, PANSS total, and PANSS negative symptoms were 0.037, 0.002, 0.002, 0.017 and 0.335, respectively. Thus, ziprasidone was statistically inferior to haloperidol on four of the five scales. The statistical reviewer also noted (page 7 of 61):

The secondary objective comparing haloperidol with ziprasidone was changed to that of comparing haloperidol with placebo **after the completion of the trial** [emphasis in original].

Thus, with respect to the original secondary objective, Trial 115 was negative. (The fact that this trial could be considered positive is because secondary outcomes are trumped by primary outcomes, which involved comparisons of ziprasidone to placebo.) Weighing the overall risk–benefit ratio of ziprasidone during the earlier review cycle, the medical review (page 3 of 223) quoted the Division Director:

“[Z]iprasidone's comparative performance (Study 115) supports a conclusion that it is less efficacious than haloperidol, a long marketed antipsychotic drug.” Dr. Leber [the Division Director] recommended a nonapprovable action….The nonapprovable letter of June 17^th^, 1998 asserted that a sufficient advantage over currently marketed antipsychotics had not been demonstrated that could outweigh the risk of potentially fatal arrhythmias because of the demonstrated QTc prolongation.

Later, a July 2000 Advisory Committee recommended that the FDA approve ziprasidone in spite of the QT issues. Following this, the inferior performance of ziprasidone relative to haloperidol ceased to be a concern, so Trial 115 could be considered, from a regulatory standpoint, supportive of efficacy. Based on this final judgment of the FDA, Trial 115 is classified as positive in Figure 1 and in our primary statistical analysis and as questionable in a post hoc analysis.

The results of another ziprasidone trial, Trial 104, were not published. Table 1 shows that, in Trial 104, two doses of ziprasidone were tested against placebo. There was also a third dose group of 10 mg/d, but it is not shown in the table or included in our analyses because that dose was not approved by the FDA. This trial involved nearly 200 patients (including the patients assigned to the low-dose group) at 17 US sites. According to the FDA statistical review (pages 16 and 12 of 61):

None of the three primary efficacy endpoints reached statistical significance at two-sided 0.05 level either based on the ITT LOCF or the Completer analyses in Study 104….There was no dose-response trend either including or excluding placebo with respect to any of the primary endpoints.

### Outcome Reporting Bias (*n* = 5 Trials)

Among the eight trials classified as having FDA-negative or -questionable outcomes (Figure 1), five were published. The FDA reviews of these five trials presented data that raised concerns about drug efficacy. Below we note whether and how these concerns were conveyed in the corresponding journal articles.

#### Iloperidone trials (*n* = 3)

One efficacy issue that was apparent from the FDA review of iloperidone, but not from the corresponding journal articles, was that the drug frequently proved to be statistically inferior to active comparators (Table 3 and detailed below).

**Table 3:** Iloperidone results (*p-*values) by trial, dose group, and diagnosis.

Trial Number/Measure	Primary Dose Group(s) (mg/d)	Schizophrenia+Schizoaffective Disorder (All Patients)		Schizophrenia Only (Post Hoc FDA Subset)		*p-*Value Highlighted in Journal Article
		Superiority versus Placebo	Inferiority versus Active Comparator	Superiority versus Placebo	Inferiority versus Active Comparator	
3000	8+12	0.065	**0.027**	0.148	0.063	**0.047** (12 mg only)
3004	10–16	**0.001**	**0.034**	0.306	**0.021**	**0.001**
3005	12–16	0.059	**<0.001**	**0.033**	**0.005**	0.09
	20–24	0.071	**0.034**	**0.005**	0.093	**0.01**
3101	24	Schizoaffective patients not recruited		**0.007**	FDA reported as statistically inferior without *p-*values	**<0.01**
Total number of positive trials		1	0	2	0	4
Total number negative or questionable trials		2	3	2	2	0
Meets FDA approval criterion of two or more positive trials		No	—	Yes	—	—

Statistically significant *p-*values (<0.05) are given in bold. Column at far right shows *p-*values from journal articles; all others are from the FDA review of iloperidone. See text for details.

Another issue apparent from the FDA review was that iloperidone's efficacy relative to placebo varied according to the patient diagnostic population studied. Consequently, as has been reported previously [33], iloperidone's path to FDA approval was somewhat convoluted. To elaborate, of the four iloperidone premarketing trials, the first three called for the recruitment of patients diagnosed with either schizophrenia or schizoaffective disorder: Trials 3000, 3004, and 3005. The protocols for these three trials were submitted to and approved by the FDA, and these trials were conducted between 1998 and 2001. The results of these three trials were presented together in a single 2008 journal article [31].

In contrast to the way these trials were reported in the journal article (details below), the FDA review initially judged only one of the three trials to be positive (Trial 3004; Table 3). Because the FDA requires two positive studies in order to approve a drug for marketing, the agency informed the drug's sponsor in 2001 that another positive trial would be required for approval. The agency added that such a trial should be restricted to patients with schizophrenia, i.e., excluding patients with schizoaffective disorder.

A fourth premarketing trial, restricted to patients with schizophrenia, was undertaken in 2005. The results from this trial were positive, and they were submitted to the FDA in a New Drug Application (NDA) in November 2007. On July 25, 2008, the agency issued a not-approvable letter, stating that the sponsor had failed to demonstrate efficacy (through two or more positive trials) in patients with schizophrenia.

After the sponsor appealed the decision, the FDA conducted a post hoc reanalysis of the first three trials, examining efficacy within the subset of patients diagnosed with schizophrenia, i.e., excluding data from the patients in those trials diagnosed with schizoaffective disorder. This reanalysis yielded a positive result for one of the initial three trials, Trial 3005 (Table 3). This positive result from Trial 3005, together with the positive result from Trial 3101, yielded a total of two positive trials in patients with schizophrenia. The FDA's criterion for approval, i.e., two or more positive trials in a defined patient population, was now achieved, thus allowing the agency to approve iloperidone in May 2009.

#### Iloperidone Trial 3000

In iloperidone Trial 3000, according to the FDA, the dose group prespecified in the protocol as primary was the combination of patients taking either 8 mg or 12 mg of iloperidone per day. For this combined dose group, using the full schizophrenia-plus-schizoaffective sample of patients, the drug–placebo difference was nonsignificant (*p* = 0.065). The corresponding journal article [31] reported this nonsignificant *p-*value in the text of the results section but preceded this with the report of a significant finding (*p* = 0.047) obtained with the 12-mg dose alone, a secondary outcome. This significant result was also reported in the abstract, while the nonsignificant result on the primary dose group was not. The FDA's findings that iloperidone was statistically inferior to haloperidol for this all-patients sample (*p* = 0.027; Table 3) was not reported in the corresponding journal article. Additionally, the FDA's finding of a lack of statistical superiority to placebo (*p* = 0.148) with the schizophrenia-only subset was not reported in the journal article [31]. However, the sponsor did not learn of the FDA's intent to focus on the schizophrenia-only subset until the not-approvable letter was issued (July 2008), some months after the journal article was published (April 2008).

#### Iloperidone Trial 3004

Among the three iloperidone trials that recruited patients diagnosed with either schizophrenia or schizoaffective disorder, Trial 3004 was the only one for which the FDA found significant results for the all-patients sample (Table 3). We have classified it as questionable for two reasons. First, although the FDA initially judged this trial to be positive based on the all-patients sample, it later conducted a post hoc analysis on the subset of patients with schizophrenia, which yielded a nonsignificant result (*p* = 0.306). Commenting on this finding, the FDA medical reviewer stated (page 110 of 247):

The results of Study 3004 do not provide evidence of efficacy of iloperidone…in the treatment of schizophrenia versus placebo over 42 days of treatment.

Second, the FDA found that iloperidone was significantly inferior to the active comparator risperidone (Table 3), both for the subset of patients with schizophrenia (*p* = 0.021) and for the all-patients group (*p* = 0.034). According to the summary review by Division Director Thomas Laughren (page 6 of 21):

Thus, either approach to defining the sample for this study yields a result that favors a standard control agent over iloperidone.

This statistical inferiority to risperidone was not reported in published version of this trial [31].

#### Iloperidone Trial 3005

Trial 3005 also yielded a mix of positive and not-positive efficacy results. As with the other iloperidone trials, the FDA review revealed evidence of iloperidone's statistical inferiority to the active comparator (Table 3). Additionally, for the all-patients (schizophrenia-plus-schizoaffective) sample, the FDA found that the 0.05 threshold for statistical significance was not achieved for either dose group, while the journal publication [31] reported a significant result (*p* = 0.01) for one of the two dose groups.

In the FDA's post hoc analysis of the subset of patients with schizophrenia, the results were significant for both dose groups (*p* = 0.033 and *p* = 0.005, respectively). Thus, as a result of the FDA's reanalysis using this patient subset, the results of Trial 3005 changed from nonsignificant to significant, the opposite of what occurred with the reanalysis of Trial 3004. As was the case for the other two trials covered in this journal article [31], the results based on this patient subset were not reported. As can be seen in Table 3, these results were favorable for Trial 3005 (though unfavorable for Trials 3000 and 3004). As stated above, the fact that Trial 3005 was positive in the schizophrenia-only subset allowed the FDA to approve iloperidone: by focusing on patients with schizophrenia and combining this result with that of Trial 3101, the FDA's requirement of two positive trials was achieved.

#### Iloperidone Trial 3101

Trial 3101 was the only iloperidone premarketing trial to include only patients with schizophrenia and exclude those with schizoaffective disorder. As shown in Table 3, iloperidone demonstrated superiority to placebo (*p* = 0.007). However, the FDA also reported that iloperidone was significantly inferior to the active comparator (ziprasidone in this trial) in most of the comparisons. (These *p-*values were not reported in the FDA review, so they do not appear in Table 3.) As with the above-mentioned trials, iloperidone's statistical inferiority to the active comparator was not reported in the corresponding journal article [34].

#### Quetiapine Trial 0006

We also classified quetiapine Trial 0006 as questionable, rather than clearly positive or negative. According to the FDA medical officer's conclusion (page 82 of 245):

On balance, this study provides marginal support for antipsychotic efficacy of quetiapine, when titrated to a wide dose range. Strictly speaking, however, the data fall short of meeting the customary level of statistical proof, particularly for the observed cases [completers] analyses.

Regarding this same trial, the statistical reviewer stated (page 2 of 40):

The borderline statistical result of the LOCF [the protocol-prespecified primary method for handling dropouts; *p* = 0.07] is likely due to the less than anticipated treatment difference at 6 weeks (−8.1 versus −2.1). Note that there was no difference at all for the completers. [For example, using the BPRS rating scale, *p* = 0.95.] Thus, Figure 5 indicates that the entire treatment difference in the LOCF analysis is due to dropouts in the first 4 weeks of the trial.

The corresponding journal article [35] communicated the results of this trial with more favorable language. The abstract opened its presentation of the results with the following:

Significant differences (p equal to or less than 0.05) between treatment groups, which favored ICI 204,636 [quetiapine], were identified throughout the trial.

The results section opened its efficacy section with the following:

On days 14, 28, and 35, when statistically significant differences between treatment groups were detected, mean changes in BPRS total scores were….

In the sentence that followed, the above-mentioned *p-*value of 0.07 was reported and described as “marginally significant and favored ICH 204,636 [quetiapine].” Following that, four *p-*values above the 0.05 significance threshold were listed and described as “marginally significant.” The results of the completers analyses, described by the FDA as showing “no difference at all” (see above), were not reported in the journal article.

#### Ziprasidone Trial 106

We have classified ziprasidone Trial 106 as neither clearly positive nor negative, but rather questionable, for the following reasons. First, the FDA medical review stated:

Because this study showed statistical significance in only two of the three primary efficacy variables in week 4 only, it merely provides fair evidence for the antipsychotic properties of ziprasidone at a dose of 60 mg bid.

The FDA's point—that the efficacy of the 120-mg dose was in question because it failed to demonstrate superiority to placebo on all primary outcomes—was not apparent in the journal article [36], which stated:

In the intent-to-treat analysis of mean changes from baseline at 4 weeks, ziprasidone 120 mg/day was significantly more effective than placebo in improving mean BPRS total and CGI-S [Clinical Global Impression Scale–Severity] scores (P<0.05).

Second, a 40-mg/d (20 mg twice daily) dose group was included in this trial, and though it demonstrated efficacy in other trials and thus became an FDA-approved dose, it did not demonstrate statistical superiority to placebo in this trial on any of the three primary variables (e.g., *p* = 0.657 on the BPRS).

In the published version of this trial [36] the nonsignificance of the results at this dose were acknowledged, but without *p-*values and, compared to the 120-mg findings, less prominently in terms of placement. In the results section, while the 120-mg/d results were mentioned in the first line of the efficacy subsection, the 40-mg results were mentioned in the ninth line. In the discussion section, while the significant 120-mg results were mentioned in the first line, the nonsignificant 40-mg results were mentioned in the middle of the third paragraph. The abstract reported several significant results for the 120-mg dose but no results for the 40-mg dose.

### Selective Reporting of Sites within Trials (*n* = 2 Trials)

#### Risperidone Trial 201

According to the FDA review, risperidone Trial 201 involved nine US sites. The total baseline sample consisted of 160 patients, of whom 156 were included in the LOCF analysis, this trial's primary method of handling dropouts. The corresponding journal article [37] reported a total sample size of only 36. The number of sites was not reported. No additional publications arising from this trial could be identified in our literature search or within a meta-analysis on risperidone [38] published four years later.

Because the FDA review did not break down Trial 201's results by site, it is unknown whether the published results were any more favorable to risperidone than those that were not published. Nevertheless, the overall results were positive according to both the FDA and the journal article. According to the FDA:

Study 201 provides unequivocal support for the effectiveness of risperidone as an antipsychotic agent.

Thus, there was evidence that the data from this trial were selectively reported in terms of the patients reported on, but this situation does not appear to meet criteria for publication bias in that it did not affect the strength and direction of the results [39], at least with respect to efficacy.

#### Risperidone Trial 204

According to the FDA review, risperidone Trial 204 was a single multicenter trial conducted at 26 sites, 20 in the US and six in Canada. Being a multicenter trial, the data from all sites were pooled in the FDA analysis. Rather than publish the results of this trial in a single journal article, the sponsor published reported the data results from the six Canadian sites in a 1993 journal article [40] as one positive trial and the data from the 20 US sites in a 1994 article [41] as a separate positive trial. The first article did not mention the US sites. The second article stated:

In the present article we report the results of a multicenter study of schizophrenic patients recruited at 20 centers in the United States. The study is part of a U.S.-Canadian collaborative investigation of risperidone in schizophrenia. The results of the Canadian arm of the investigation have been published [citation referencing [42]].

Three years later, the drug's sponsor reported the US and Canadian sites' results in a single article [42], but the article's first sentence stated:

Two pivotal controlled trials of risperidone have been conducted in North America, the Canadian study of Chouinard et al [citation referencing [40]] and the United States study of Marder and Meibach [citation referencing [41]]. In this report, we present the results of an analysis of the combined data from the two trials….

Therefore, while the FDA was aware that this was a single trial and analyzed it as such, readers, editors, and reviewers of journal articles would have thought they were reading two different trials. This “split” is shown with a horizontal dotted line in Figure 1, and it contributed to the lack of one-to-one correspondence between the FDA studies and journal articles shown in Table 1.

## Discussion

### Recapitulation of Findings

These data provide mixed evidence for publication bias for antipsychotic drug trials. One-sixth (17%) of the trials were unpublished. Among the four unpublished trials, three failed to show that the drug was superior to placebo, and one showed that the drug was statistically inferior to the active comparator. Among the 20 published trials, 15 (two-thirds) were FDA-positive; the five that were not positive showed some evidence of outcome reporting bias. However, the association between trial outcome and publication status was not statistically significant. The mean effect size derived from the published literature was only slightly higher than that derived from the FDA reviews, and the difference between them did not reach statistical significance. On the other hand, within the FDA dataset, the mean effect size of the published trials was approximately double that of the unpublished trials.

### Publication Bias and Antipsychotics

Previous work on publication bias among antipsychotic trials has focused on alleged advantages of specific antipsychotics over one another [43],[44]. And within the context of a meta-analysis of second-generation antipsychotics, small study bias was suggested by an asymmetric funnel plot [45]. In the present study, FDA data provided a relatively unbiased control dataset with which to measure the influence of publication bias on apparent drug efficacy. Our approach is perhaps best compared to two previous studies that employed drug regulatory data to examine publication bias for antidepressants: one by our group, which used FDA data and similar methodology [8], and an earlier study, which used data from the Swedish drug regulatory authority [46].

### Publication Bias for Antipsychotics versus Antidepressants

Compared to the findings for antidepressants, these findings for antipsychotics are less striking. (The overall results for the two drug classes are compared at the bottom of Figure 3.) We believe this is fundamentally related to the larger FDA-based effect size for antipsychotics compared to antidepressants [8], which increases the probability that, in any given trial, the drug–placebo difference will reach statistical significance. Indeed, two-thirds of the antipsychotic trials were positive, compared to one-half of the antidepressant trials [8]. With a larger proportion of positive trials, it should not be surprising that a larger proportion of the antipsychotic trials were published. We do not know whether this is due to a higher manuscript submission rate by drug companies and their investigators, a higher acceptance rate by journals, or both.

As a further consequence of the increased proportion of positive trials, fewer trials were needed, on a per-drug basis, to attain the two positive trials required for FDA approval (three here versus approximately six for antidepressants). This, combined with the fact that we were working with fewer drugs (eight here versus 12 for antidepressants), led to a smaller total number of trials (24 versus 74) and thereby less statistical power. In the meta-analyses, the smaller *n* associated with the unpublished negative and questionable trials carried little meta-analytic weight relative to the larger *n* associated with the positive and published trials. This diluted the impact of the unpublished trials when the overall effect size was calculated, leading to a smaller gap between the FDA- and journal-based overall effect size values (8% here versus 32% for antidepressants).

Comparing drug classes, the published literature suggests that the effect size for antipsychotics is only slightly greater than that for antidepressants, but FDA data reveal that the effect size gap between these two drug classes is much larger (Figure 3). A similar discrepancy between published and FDA data can be seen when one compares the proportion of positive trials for the two drug classes. Examined either way, publication bias can blur distinctions between effective and ineffective drugs.

### Effect Size in Context

The overall effect size we found, 0.44, was somewhat lower than those from two previous meta-analyses that also made use of FDA data from placebo-controlled trials, probably because of methodological differences. One of these meta-analyses [45] reported an overall effect size of 0.51. It covered somewhat different drugs and comingled (limited) FDA data with published data, while we deliberately kept these two data sources separate in order to contrast them. The other meta-analysis [47] reported an effect size of 0.53. It was based on FDA data exclusively, but it covered only three of the eight drugs in the present study. That meta-analysis was published before FDA data became available on the two drugs found in this study to have the lowest effect sizes, ziprasidone (0.30) and iloperidone (0.28).

Because the effect size point estimate of 0.44 is less than 0.5, there is the risk that some parties will declare that antipsychotics have failed a critical litmus test for clinical significance, as has been declared for antidepressants [48],[49]. Space does not permit a full discussion as to why this reasoning is problematic [50], but we will touch briefly on some of the issues. (1) This 0.5 cutoff seems to derive from Cohen's [51] suggestion that 0.5 be used as a landmark for a medium effect, but Cohen never mentioned clinical significance nor, for that matter, clinical trials. (2) In proposing these landmarks, Cohen cautioned, “The values chosen had no more reliable a basis than my own intuition.” Put another way, they are not evidence-based. (3) It seems simplistic to think that one cutoff should apply uniformly across all fields of scientific inquiry, including all classes of drugs. Rather, as has been stated, “The most challenging and urgent task remains unsolved: developing the principles that underlie the thresholds of clinical significance in different clinical contexts” [52]. (4) Because of publication bias, effect sizes derived from the published literature should be interpreted with some caution. Some effect sizes that appear to exceed 0.5 might need to be revised downward once they are recalculated using data that are less vulnerable to publication bias, such as FDA data. Until such time, considering the evidence that publication bias is pervasive throughout medicine [3], we believe that any decisions on thresholds for clinical significance are premature.

### Limitations

Lack of statistical power, due to the small number of trials analyzed (see above), was a major limitation of this study. Because of this, some of the statistically nonsignificant results could represent type II error (false negatives). Further, the statistical test for trial outcome versus publication status examined whether trials were published but not how they were published (outcome reporting bias).

Although the forest plots might seem to suggest significant between-drug differences, this study was not designed to address comparative effectiveness. The degree of heterogeneity is unclear: the 95% confidence intervals around *I* ^2^ were wide, as appears to be the case in the majority of meta-analyses of medical interventions [29].

While this study addresses the efficacy of antipsychotics, it does not address their safety, an integral component of the clinician's risk–benefit analysis, nor their “real world” effectiveness [53]. In the trials we studied, efficacy was measured using scales that give little weight to disabling cognitive and negative symptoms [54]. These data apply only to adult, not pediatric or geriatric, patients. And they apply only to schizophrenia and schizoaffective disorder, not to other conditions for which these drugs are used.

This study assumes that the FDA database serves as a gold standard that is complete and unbiased, but caveats must be acknowledged. Drug companies must register trials with the FDA before they can begin them, but this applies only if the drug company is already pursuing marketing authorization in the US. Sometimes drug companies conduct clinical trials programs and obtain marketing approval outside the US and only approach the FDA at a later point in time. In such cases it is conceivable that the FDA, having not had the benefit of a priori trial registration, might not learn about the existence of certain non-US trials. A second caveat arises from the fact that what the FDA routinely makes publicly available, the drug approval packages [5],[11], contain data primarily from premarketing trials. Postmarketing trials are generally omitted, and these may represent the majority of the trials conducted on a given drug.

### Implications

Selective reporting of research results undermines the integrity of the evidence base, which ultimately deprives clinicians of accurate data for prescribing decisions. With further studies investigating publication bias in other drug classes, a more accurate evidence base can emerge. To that end, increased access to FDA reviews has been advocated [5],[55]. At the present time, the FDA is not as transparent with its clinical trial data as it could be. For example, we pointed out in 2004 that the reviews for several antipsychotic drugs were posted on the FDA web site, but only for the original indication of schizophrenia and not for bipolar mania [5]. More than seven years later, the mania reviews remain inaccessible. On the other hand, it is encouraging that the FDA has convened a Transparency Task Force [56]. If the agency fulfills its mission to increase transparency, the public health will surely benefit.

## Supporting Information

Table S1 **Two-by-two tables of trial outcome according to the FDA versus publication status.**
(DOC)

Table S2 **Meta-analysis of clinical trial data from FDA drug approval packages—output from statistical program Stata.**
(DOC)

Table S3 **Meta-analysis of clinical trial data from the published literature.** Output from statistical program Stata.
(DOC)

Text S1 **PRISMA checklist.**
(DOC)

Text S2 **Supplemental methods and results.**
(DOC)

## Abbreviations

BPRS: Brief Psychiatric Rating Scale
CI_95%_: 95% confidence interval
FDA: US Food and Drug Administration
LOCF: last observation carried forward
PANSS: Positive and Negative Syndrome Scale
risperidone LAI: risperidone long-acting injection
