# Supplementary figures and images for: Publication Bias in Antipsychotic Trials: An Analysis of Efficacy Comparing the Published Literature to the US Food and Drug Administration Database
Source: PLoS Med. 2012 Mar 20;9(3):e1001189. doi: 10.1371/journal.pmed.1001189 (PMC3308934; doi:10.1371/journal.pmed.1001189)

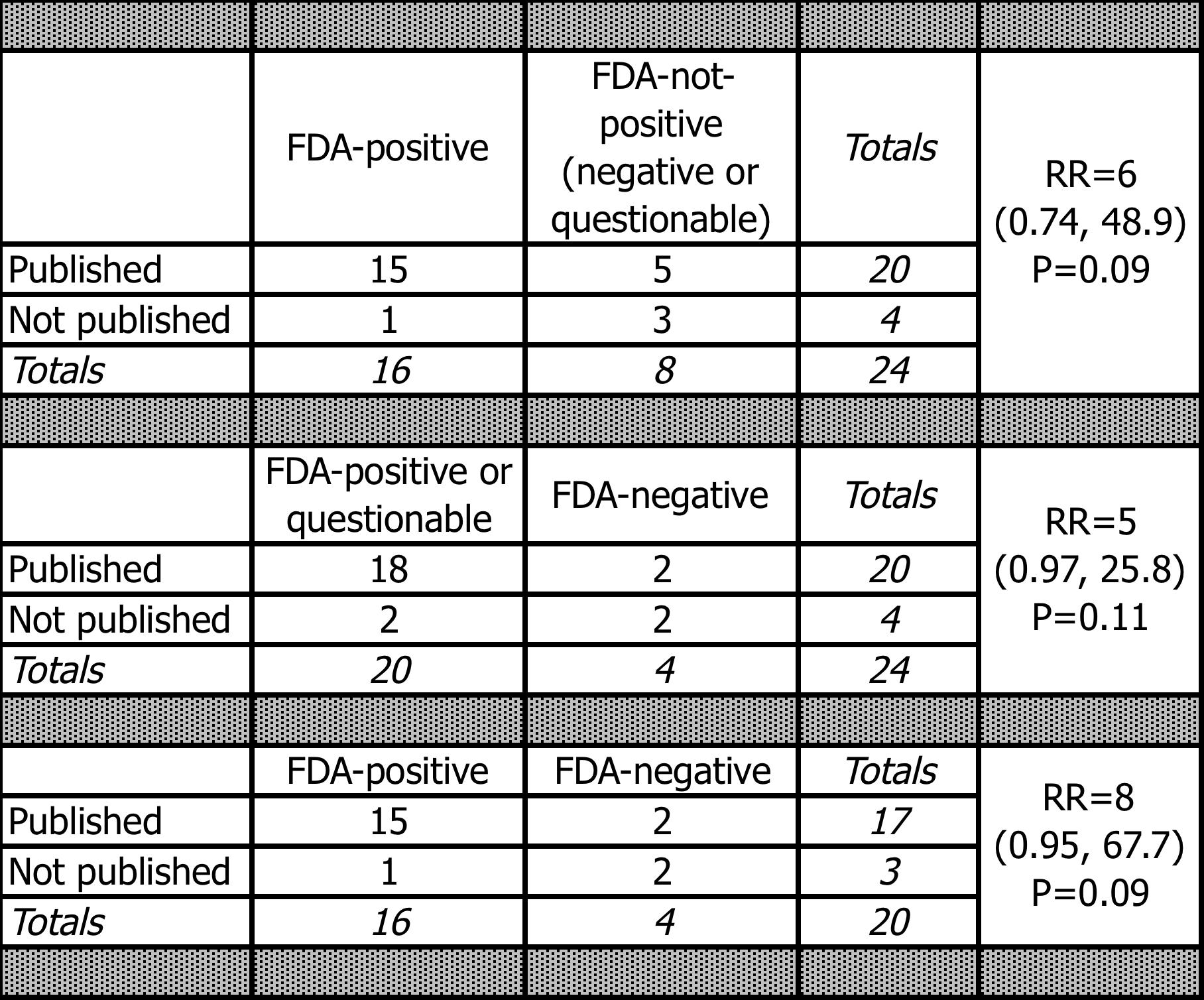


Table S1. Trial outcome versus publication status.

Supplement: Table S1 — Two-by-two tables of trial outcome according to the FDA versus publication status. (DOC) [file pmed.1001189.s001.doc]
