# Supplementary material for: Publication Bias in Antipsychotic Trials: An Analysis of Efficacy Comparing the Published Literature to the US Food and Drug Administration Database
Source: PLoS Med. 2012 Mar 20;9(3):e1001189. doi: 10.1371/journal.pmed.1001189 (PMC3308934; doi:10.1371/journal.pmed.1001189)
Supplement: Table S2 — Meta-analysis of clinical trial data from FDA drug approval packages—output from statistical program Stata. (DOC) [file pmed.1001189.s002.doc]

**Table S2. Meta-analysis of FDA data—Stata® output**

. metan g se if source==1, label(namevar=study) by(drug) random

Study | ES [95% Conf. Interval] % Weight

---------------------+---------------------------------------------------

aripiprazole

93202 | 0.330 -0.148 0.809 1.85

97201 | 0.463 0.221 0.705 4.69

97202 | 0.442 0.201 0.684 4.69

138001 | 0.489 0.266 0.712 5.09

94202 | 0.163 -0.160 0.486 3.32

Sub-total |

D+L pooled ES | 0.415 0.294 0.536 19.64

---------------------+---------------------------------------------------

iloperidone

3101 | 0.280 0.076 0.483 5.54

3000 (SCZ and SA) | 0.261 0.003 0.520 4.36

3004 (SCZ and SA) | 0.382 0.154 0.610 4.98

3005 (SCZ and SA) | 0.233 0.043 0.422 5.88

Sub-total |

D+L pooled ES | 0.284 0.176 0.392 20.75

---------------------+---------------------------------------------------

olanzapine

HGAD | 0.628 0.318 0.938 3.50

HGAP | 0.502 0.100 0.904 2.43

Sub-total |

D+L pooled ES | 0.581 0.335 0.827 5.93

---------------------+---------------------------------------------------

paliperidone

303 | 0.695 0.489 0.901 5.46

304 | 0.418 0.183 0.652 4.84

305 | 0.694 0.482 0.905 5.35

Sub-total |

D+L pooled ES | 0.610 0.436 0.783 15.65

---------------------+---------------------------------------------------

quetiapine

0001/0008 | 0.358 0.108 0.607 4.53

0006 | 0.354 -0.030 0.738 2.61

0013 | 0.564 0.253 0.875 3.49

Sub-total |

D+L pooled ES | 0.421 0.248 0.595 10.63

---------------------+---------------------------------------------------

risp_depot

Ris-USA-121 | 0.579 0.325 0.832 4.46

Sub-total |

D+L pooled ES | 0.579 0.325 0.832 4.46

---------------------+---------------------------------------------------

risperidone

201 | 0.660 0.265 1.055 2.50

204-Canada sites | 0.843 0.347 1.339 1.75

204-US sites | 0.734 0.443 1.025 3.79

Sub-total |

D+L pooled ES | 0.732 0.521 0.944 8.04

---------------------+---------------------------------------------------

ziprasidone

104 | 0.034 -0.312 0.379 3.03

106 | 0.288 -0.070 0.647 2.88

114 | 0.405 0.156 0.654 4.55

115 | 0.346 0.092 0.600 4.45

Sub-total |

D+L pooled ES | 0.301 0.154 0.448 14.90

---------------------+---------------------------------------------------

Overall |

D+L pooled ES | 0.441 0.369 0.513 100.00

---------------------+---------------------------------------------------

Test(s) of heterogeneity:

Heterogeneity degrees of

statistic freedom P I-squared** Tau-squared

aripiprazole 3.08 4 0.545 0.0% 0.0000

iloperidone 1.03 3 0.795 0.0% 0.0000

olanzapine 0.24 1 0.626 0.0% 0.0000

paliperidone 3.82 2 0.148 47.7% 0.0112

quetiapine 1.18 2 0.554 0.0% 0.0000

risp_depot 0.00 0 . .% 0.0000

risperidone 0.32 2 0.852 0.0% 0.0000

ziprasidone 3.09 3 0.377 3.0% 0.0007

Overall 41.86 24 0.013 42.7% 0.0138

** I-squared: the variation in ES attributable to heterogeneity)

Note: between group heterogeneity not calculated;

only valid with inverse variance method

Significance test(s) of ES=0

aripiprazole z= 6.72 p = 0.000

iloperidone z= 5.17 p = 0.000

olanzapine z= 4.64 p = 0.000

paliperidone z= 6.90 p = 0.000

quetiapine z= 4.76 p = 0.000

risp_depot z= 4.48 p = 0.000

risperidone z= 6.78 p = 0.000

ziprasidone z= 4.01 p = 0.000

Overall z= 11.96 p = 0.000

-------------------------------------------------------------------------
