# Supplementary material for: Publication Bias in Antipsychotic Trials: An Analysis of Efficacy Comparing the Published Literature to the US Food and Drug Administration Database
Source: PLoS Med. 2012 Mar 20;9(3):e1001189. doi: 10.1371/journal.pmed.1001189 (PMC3308934; doi:10.1371/journal.pmed.1001189)
Supplement: Table S3 — Meta-analysis of clinical trial data from the published literature. Output from statistical program Stata. (DOC) [file pmed.1001189.s003.doc]

**Table S3. Meta-analysis of journal data—Stata® output**

. metan g se if source==2, label(namevar=study) by(drug) random

Study | ES [95% Conf. Interval] % Weight

---------------------+---------------------------------------------------

aripiprazole

97201 | 0.455 0.218 0.693 5.57

97202 | 0.447 0.206 0.689 5.47

138001 | 0.490 0.267 0.713 5.92

Sub-total |

D+L pooled ES | 0.466 0.331 0.600 16.96

---------------------+---------------------------------------------------

iloperidone

3101 | 0.280 0.076 0.483 6.42

3000 (SCZ and SA) | 0.261 0.003 0.520 5.10

3004 (SCZ and SA) | 0.382 0.154 0.610 5.79

3005 (SCZ and SA) | 0.233 0.043 0.422 6.80

Sub-total |

D+L pooled ES | 0.284 0.176 0.392 24.11

---------------------+---------------------------------------------------

olanzapine

HGAD | 0.640 0.330 0.951 4.12

HGAP | 0.600 0.195 1.005 2.87

Sub-total |

D+L pooled ES | 0.625 0.379 0.872 6.99

---------------------+---------------------------------------------------

paliperidone

303 | 0.695 0.488 0.901 6.34

304 | 0.417 0.183 0.652 5.64

305 | 0.694 0.482 0.905 6.21

Sub-total |

D+L pooled ES | 0.609 0.436 0.783 18.19

---------------------+---------------------------------------------------

quetiapine

0001/0008 | 0.349 0.098 0.601 5.25

0006 | 0.348 -0.030 0.726 3.16

0013 | 0.564 0.253 0.875 4.12

Sub-total |

D+L pooled ES | 0.416 0.242 0.590 12.53

---------------------+---------------------------------------------------

risp_depot

Ris-USA-121 | 0.557 0.304 0.810 5.22

Sub-total |

D+L pooled ES | 0.557 0.304 0.810 5.22

---------------------+---------------------------------------------------

risperidone

201 | 1.495 0.590 2.400 0.72

204-Canada sites | 0.843 0.347 1.339 2.09

204-US sites | 0.734 0.443 1.025 4.46

Sub-total |

D+L pooled ES | 0.842 0.544 1.140 7.27

---------------------+---------------------------------------------------

ziprasidone

106 | 0.273 -0.086 0.631 3.41

114 | 0.405 0.156 0.654 5.32

Sub-total |

D+L pooled ES | 0.362 0.158 0.566 8.73

---------------------+---------------------------------------------------

Overall |

D+L pooled ES | 0.476 0.396 0.556 100.00

---------------------+---------------------------------------------------

Test(s) of heterogeneity:

Heterogeneity degrees of

statistic freedom P I-squared** Tau-squared

aripiprazole 0.08 2 0.963 0.0% 0.0000

iloperidone 1.03 3 0.795 0.0% 0.0000

olanzapine 0.02 1 0.877 0.0% 0.0000

paliperidone 3.83 2 0.147 47.8% 0.0112

quetiapine 1.26 2 0.532 0.0% 0.0000

risp_depot 0.00 0 . .% 0.0000

risperidone 2.48 2 0.290 19.3% 0.0158

ziprasidone 0.35 1 0.552 0.0% 0.0000

Overall 36.89 20 0.012 45.8% 0.0150

** I-squared: the variation in ES attributable to heterogeneity)

Note: between group heterogeneity not calculated;

only valid with inverse variance method

Significance test(s) of ES=0

aripiprazole z= 6.77 p = 0.000

iloperidone z= 5.17 p = 0.000

olanzapine z= 4.98 p = 0.000

paliperidone z= 6.88 p = 0.000

quetiapine z= 4.69 p = 0.000

risp_depot z= 4.31 p = 0.000

risperidone z= 5.54 p = 0.000

ziprasidone z= 3.47 p = 0.001

Overall z= 11.71 p = 0.000

-------------------------------------------------------------------------
