# Supplementary material for: Publication Bias in Antipsychotic Trials: An Analysis of Efficacy Comparing the Published Literature to the US Food and Drug Administration Database
Source: PLoS Med. 2012 Mar 20;9(3):e1001189. doi: 10.1371/journal.pmed.1001189 (PMC3308934; doi:10.1371/journal.pmed.1001189)
Supplement: Text S2 — Supplemental methods and results. (DOC) [file pmed.1001189.s005.doc]

**Text S2: Supplemental Methods and Results**

Methods, supplemental

FDA reviews: procurement and selection

Although the clinical trial programs studied here led to FDA approval for the indication of schizophrenia, 57% (13/23) of the trials also included patients with schizoaffective disorder and/or schizophreniform disorder, along with patients with schizophrenia.

Three recently approved antipsychotics could not be included because the FDA had not posted their reviews as of our cutoff date of September 2009: (1) asenapine (Saphris, FDA-approved August 2009), (2) quetiapine extended release (Seroquel XR®, FDA-approved May 2007), and (3) paliperidone extended release injectable suspension (Invega Sustenna®, FDA-approved July 2009).

As stated in the main text, we employed data on trials obtained from Drug Approval Packages, within which we looked for data on trials that the FDA considered relevant to its determination of drug efficacy. We thus excluded two trials that were included in meta-analyses that made use of FDA data [1,2], as detailed in the following paragraphs.

One of these trials, risperidone trial 72, was excluded because it was not part of the initial drug approval package. Three years after the original approval of risperidone, which was based on twice-daily dosing, the drug’s sponsor submitted risperidone trial 72 to support a change in labeling to allow for once-daily dosing. Because this was a postmarketing trial and reviewed as part of a supplemental application to the FDA, rather than as part of the initial drug approval package, it was excluded from our study.

The other trial, quetiapine trial 0004, was mentioned briefly in the reviews within the drug approval package, but the FDA did not consider it relevant to its determination of drug efficacy. That is because the FDA requires demonstration of drug efficacy in at least two “adequate and well controlled trials” [3], whereas this trial’s total sample size was only 12. It was referred to in the medical review as a “small pilot trial” that “will not be considered in detail in this review.” In contrast to the trials considered relevant to efficacy, no P values, mean changes, standard deviations, standard errors, or confidence intervals were reported. Although this trial was listed in the Table of All Studies, it was classified as a pharmacokinetic trial rather than a placebo-controlled phase 2/3 trial. For these reasons we excluded quetiapine trial 0004 from our study.

Further literature search for unpublished trials

Aripiprazole trials. The search within 3 recent review articles did not lead to any publications for aripiprazole trials 93202 and 94202. One review article on aripiprazole [4] referenced 2 publications [5,6] that we had already matched to trials 97201 and 97202, respectively, and 3 conference posters, which did not meet our criteria for ‘published’. A second review article [7] cited the same two publications [5,6]. A third review article [8], aside from referencing journal publications already known to us, referenced only the FDA review of aripiprazole and one conference abstract. Finally we consulted the sponsor’s web page for clinical trial results [9]. The trial numbers listed all began with the numbers “138”. Thus, among the FDA-registered trials included in our study, only Trial 138001 was listed. Therefore we concluded that aripiprazole Studies 93202 and 94202 were unpublished.

Ziprasidone trials. The search within 3 recent review articles did not lead to any publications for ziprasidone trials 104 and 115. In one review article on ziprasidone [10], there were only two listed short-term placebo-controlled trials. Both were published and had already been located by us and linked to FDA-registered trials, studies 114 and 106, respectively [11,12]. In a second review article [13], ziprasidone trials 104 and 115 were referred to in Figure 5 of that article as “Study 104 in Pfizer document” and “Study 115 in Pfizer document”, respectively. The referenced Pfizer document was the Advisory Committee information on the FDA website [14], which provided no publication information. A third review article on ziprasidone [15] handled studies 104 and 115 in the same way, i.e. by referencing the FDA Advisory Committee documents. An additional fourth review article [16] handled them by referencing the FDA review documents. As a final check, we consulted the sponsor’s internet postings of clinical trial results [17], but we found no record of any of the FDA-registered trials included in our study. We therefore concluded that Studies 104 and 115 were unpublished.

Data extraction

Doses included

As stated in the main text, we included data pertaining only to dosages later approved as safe and effective according to the original product labeling. The FDA-approved dose ranges, in alphabetical order by generic drug name, are: aripiprazole 10-30 mg/day; iloperidone 12-24 mg/day; olanzapine 10-15 mg/day; paliperidone 3-12 mg/day; quetiapine 150-750 mg/day; risperidone (immediate release oral tablet) 4-16 mg/day; risperidone (long-acting injection) 25-50 mg intramuscular every 2 weeks; and ziprasidone 40-200 mg/day.

Selection of rating scale

The main text states, “For all but one study, the primary scales according to the two data sources (FDA reviews and the journal articles) matched.” The trial referred to here was ziprasidone trial 114, for which the FDA designated the BPRS as one of the primary scales and did not present data from the PANSS, while the journal presented the PANSS first.

Determination of FDA regulatory decision

Questionable trials included failed trials [18], trials with a mix of positive and negative primary outcomes, trials that were nonsignificant on the primary outcome but positive on several secondary outcomes, trials about which, in our opinion, the FDA reviewer(s) expressed doubt whether the outcome was positive, and trials for which the FDA shifted its conclusion regarding outcome (esp. with iloperidone). For fixed-dose (multiple dose) studies with a mix of significant and nonsignificant results on different dosages, we used the FDA’s stated overall decisions on the studies. As stated in the main text, when we deemed the judgment in the FDA’s review unclear, we sought clarification from the original product labeling. This is the labeling made public at the time of approval, and it is available from the FDA website.

Results, supplemental

Trial outcome versus publication status

Within Table S1 are three 2-by-2 sub-tables. They differ in how the trials in the Questionable category were handled. In the first sub-table, which corresponds to the analysis presented in the main text, the FDA-questionable trials are combined with the FDA-negative trials into a not-positive category. In the second, they are combined with the FDA-positive trials. In the third, they are excluded. As can be seen, the three risk ratio (RR) values are similar, as are the resulting values for Fisher Exact P. Statistical significance was not achieved in any of these analyses.

Supplemental results from meta-analysis

Output from statistical program

The output shown in the following pages was generated by metan, the meta-analytic program within Stata®. This output provides details not included in the main text. Meta-analysis of the data from the FDA is shown in Table S2; meta-analysis of data from the corresponding journal articles is shown in Table S3. As these tables show, both datasets showed significant heterogeneity (43% and 46%, respectively) arising from the paliperidone trials (48% for both datasets). The journal dataset also some showed heterogeneity within the risperidone trials (19%). Otherwise, for the other drugs and within both datasets, heterogeneity was negligible (0% to 3%).

Results on iloperidone by diagnostic group(s) included in meta-analysis

The effect size for iloperidone differed slightly depending the patients included in the analysis. Including all patients recruited, i.e. those diagnosed with either schizophrenia or schizoaffective disorder, iloperidone’s effect size, whether based on the FDA or the journal data, was 0.284 (CI_95%_ 0.176 to 0.392, Z=5.17, P<0.0005). As noted in the main text and elsewhere in this appendix, the FDA conducted analyses on the subset of patients diagnosed with schizophrenia, i.e. excluding the data from patients diagnosed with schizoaffective disorder. (There was no journal-based effect size for this subset of patients, since the journal article [19] reported only the results for both diagnoses combined.) Using these data, the FDA-based effect size was 0.264 (CI_95%_ 0.145 to 0.383, Z=4.36, P<0.0005). Thus the FDA’s exclusion of patients diagnosed with schizoaffective disorder led to a small decrease in effect size of 0.02. In the main text, the iloperidone meta-analytic results presented are based on the all-patients sample.

Relationship between baseline scores and effect size values

Unlike what has been reported with antidepressants [20,21], we found no suggestion of a linear relationship between baseline scores and effect size.

References

1. Leucht S, Arbter D, Engel RR, Kissling W, Davis JM (2009) How effective are second-generation antipsychotic drugs? A meta-analysis of placebo-controlled trials. Mol Psychiatry 14: 429-447.

2. Woods SW, Stolar M, Sernyak MJ, Charney DS (2001) Consistency of atypical antipsychotic superiority to placebo in recent clinical trials. Biol Psychiatry 49: 64-70.

3. Food and Drug Administration, Center for Drug Evaluation and Research (1998) Guidance for Industry: Providing Clinical Evidence of

Effectiveness for Human Drug and Biological Products. pp. 6, 9.

4. DeLeon A, Patel NC, Crismon ML (2004) Aripiprazole: a comprehensive review of its pharmacology, clinical efficacy, and tolerability. Clin Ther 26: 649-666.

5. Kane JM, Carson WH, Saha AR, McQuade RD, Ingenito GG, et al. (2002) Efficacy and safety of aripiprazole and haloperidol versus placebo in patients with schizophrenia and schizoaffective disorder. J Clin Psychiatry 63: 763-771.

6. Potkin SG, Saha AR, Kujawa MJ, Carson WH, Ali M, et al. (2003) Aripiprazole, an antipsychotic with a novel mechanism of action, and risperidone vs placebo in patients with schizophrenia and schizoaffective disorder. Arch Gen Psychiatry 60: 681-690.

7. Swainston Harrison T, Perry CM (2004) Aripiprazole: a review of its use in schizophrenia and schizoaffective disorder. Drugs 64: 1715-1736.

8. El-Sayeh HG, Morganti C, Adams CE (2006) Aripiprazole for schizophrenia. Systematic review. The British Journal of Psychiatry 189: 102-108.

9. Bristol-Myers Squibb (2010) Clinical Trial Results: Abilify.

10. Greenberg WM, Citrome L (2007) Ziprasidone for schizophrenia and bipolar disorder: a review of the clinical trials. CNS Drug Rev 13: 137-177.

11. Daniel DG, Zimbroff DL, Potkin SG, Reeves KR, Harrigan EP, et al. (1999) Ziprasidone 80 mg/day and 160 mg/day in the acute exacerbation of schizophrenia and schizoaffective disorder: a 6-week placebo-controlled trial. Ziprasidone Study Group. Neuropsychopharmacology 20: 491-505.

12. Keck P, Buffenstein A, Ferguson J, Feighner J, Jaffe W, et al. (1998) Ziprasidone 40 and 120 mg/day in the acute exacerbation of schizophrenia and schizoaffective disorder: a 4-week placebo-controlled trial. Psychopharmacology (Berl) 140: 173-184.

13. Matza LS, Baker TM, Revicki DA (2005) Efficacy of olanzapine and ziprasidone for the treatment of schizophrenia: a systematic review. CNS Drugs 19: 499-515.

14. US Food and Drug Administration, Center for Drug Evaluation and Research, FDA Psychopharmacological Drugs Advisory Committee (19 July 2000) Briefing Document for ZELDOX® CAPSULES (Ziprasidone HCl),. Pfizer.

15. Swainston Harrison T, Scott LJ (2006) Ziprasidone: a review of its use in schizophrenia and schizoaffective disorder. CNS Drugs 20: 1027-1052.

16. Woods SW, Gueorguieva RV, Baker CB, Makuch RW (2005) Control group bias in randomized atypical antipsychotic medication trials for schizophrenia. Arch Gen Psychiatry 62: 961-970.

17. ClinicalStudyResults.org (2010) Clinical Trial Results: Geodon.

18. Otto MW, Nierenberg AA (2002) Assay sensitivity, failed clinical trials, and the conduct of science. Psychother Psychosom 71: 241-243.

19. Potkin SG, Litman RE, Torres R, Wolfgang CD (2008) Efficacy of iloperidone in the treatment of schizophrenia: initial phase 3 studies. J Clin Psychopharmacol 28: S4-11.

20. Kirsch I, Deacon BJ, Huedo-Medina TB, Scoboria A, Moore TJ, et al. (2008) Initial severity and antidepressant benefits: a meta-analysis of data submitted to the Food and Drug Administration. PLoS Med 5: e45.

21. Fournier JC, DeRubeis RJ, Hollon SD, Dimidjian S, Amsterdam JD, et al. (2010) Antidepressant drug effects and depression severity: a patient-level meta-analysis. JAMA 303: 47-53.
